# Moderate-Intensity Exercise Affects Gut Microbiome Composition and Influences Cardiac Function in Myocardial Infarction Mice

**DOI:** 10.3389/fmicb.2017.01687

**Published:** 2017-09-01

**Authors:** Zuheng Liu, Hai-Yue Liu, Haobin Zhou, Qiong Zhan, Wenyan Lai, Qingchun Zeng, Hao Ren, Dingli Xu

**Affiliations:** ^1^State Key Laboratory of Organ Failure Research, Department of Cardiology, Nanfang Hospital, Southern Medical University Guangzhou, China; ^2^Key Laboratory for Organ Failure Research, Ministry of Education of the People’s Republic of China Guangzhou, China; ^3^Department of Environmental Health, School of Public Health, Southern Medical University Guangzhou, China; ^4^Department of Rheumatology, Nanfang Hospital, Southern Medical University Guangzhou, China

**Keywords:** exercise, gut microbiome, cardiac function, myocardial infarction, 16S rRNA

## Abstract

Physical exercise is commonly regarded as protective against cardiovascular disease (CVD). Recent studies have reported that exercise alters the gut microbiota and that modification of the gut microbiota can influence cardiac function. Here, we focused on the relationships among exercise, the gut microbiota and cardiac function after myocardial infarction (MI). Four-week-old C57BL/6J mice were exercised on a treadmill for 4 weeks before undergoing left coronary artery ligation. Cardiac function was assessed using echocardiography. Gut microbiomes were evaluated post-exercise and post-MI using 16S rRNA gene sequencing on an Illumina HiSeq platform. Exercise training inhibited declines in cardiac output and stroke volume in post-MI mice. In addition, physical exercise and MI led to alterations in gut microbial composition. Exercise training increased the relative abundance of *Butyricimonas* and *Akkermansia*. Additionally, key operational taxonomic units were identified, including 24 lineages (mainly from Bacteroidetes, *Barnesiella, Helicobacter, Parabacteroides, Porphyromonadaceae, Ruminococcaceae*, and *Ureaplasma*) that were closely related to exercise and cardiac function. These results suggested that exercise training improved cardiac function to some extent in addition to altering the gut microbiota; therefore, they could provide new insights into the use of exercise training for the treatment of CVD.

## Introduction

Exercise has been associated with the risk of cardiovascular disease (CVD) for many years (Mitka, 2013). Recently, studies have revealed that physical exercise influences the gut microbiome (Petriz et al., 2014; Denou et al., 2016). For example, Allen et al. (2015) reported that forced and voluntary exercise had different effects on the intestinal microbiota and were associated with different clinical outcomes in inflammatory bowel disease (IBD). Another research study implied that athletes have a microbiome profile that is distinct from that of the general population (Clark and Mach, 2016). Even short-term exercise challenge can alter the gut microbiome in certain diseases (Shukla et al., 2015).

Furthermore, the gut microbiota has also been shown to play pivotal roles in the initiation and progression of CVD (Tang et al., 2017). Previous studies have reported that gut bacteria participate in the formation and deterioration of atherosclerosis by changing the composition of the intestinal microbiota and producing microbial metabolites, such as trimethylamine-N-oxide (TMAO), and other studies have indicated that TMAO is increased in heart failure patients and positively correlated with its severity (Kitai et al., 2016; Organ et al., 2016; Suzuki et al., 2016). In addition, it was reported that TMAO was a prognostic marker of cardiovascular events after acute coronary syndrome (Fernandez-Ruiz, 2017), and the microbiome composition was found to be different between heart failure patients and healthy controls (Tang et al., 2017; Zabell and Tang, 2017). The results of these studies collectively indicate that the gut microbiota might be an attractive target for therapies for CVD and might provide a novel potential mechanism for how exercise prevents heart disease.

Both the gut microbiome and exercise have been implicated in heart disease. However, the effect of exercise training on the microbiome in myocardial infarction (MI) remains unexplored. Here, we hypothesize that exercise training will trigger changes in the gut microbiome that favor a healthier phenotype and improve cardiac function in MI mice. A potential mechanism by which exercise and MI might influence the gut microbiota involves changes in hemodynamics and intestinal blood perfusion, which alter the microenvironment in which gut bacteria reside. These bacteria subsequently influence the endocrine or immune system and thereby affect cardiac function. In this study, we explore the relationships among exercise, the gut microbiota and MI. These results suggest a potential new strategy by which exercise benefits the cardiovascular system by altering the microbiome.

## Materials and Methods

### Experimental Animals

**FIGURE 1:**
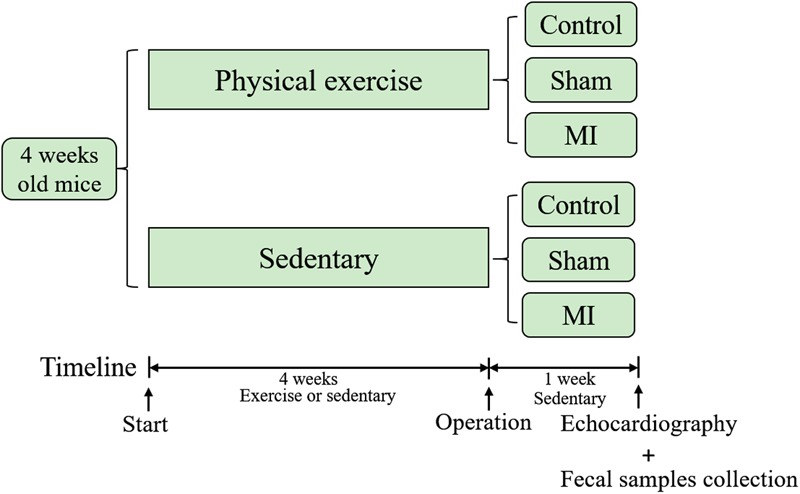
Timeline of the study. Mice were subjected to exercise or allowed to remain sedentary in cages for 4 weeks before they underwent a coronary artery ligation or sham operation. Fecal samples were collected and echocardiography was performed 1 week after the operation.

The timeline of this study is illustrated in **Figure 1**. Four-week-old male C57BL/6J mice were obtained from the laboratory of the animal center of Southern Medical University and used in this study. The breeding and housing conditions of the experimental animals complied with the People’s Republic of China National Standards (GB14925-2010). The living environment of the mice had good sealing, permeability, ventilation and lighting. Animal housing was maintained at 20–26°C with a relative humidity of 40–70%. The air was cycled a minimum of ≥10 times/hour, the ammonia concentration was ≤14 mg/mł, noise levels were kept at ≤60 dB (A), and illumination ranged from 100 to 200 lx. All mice were housed in cages under a 12-h light-dark cycle. Food and water were freely available to the mice, and care was taken to ensure that the drinking water met the requirement of the People’s Republic of China National Standards (GB5749). The mice were randomly assigned to the treadmill running group (athletic group) or the caged control group. The athletic mice were run on a treadmill system (Biowill, Shanghai) for 30 min/day at 12–15 m per minute throughout a 4-week period, while the corresponding control mice were allowed to be sedentary in their cages. After 4 weeks of exercise training, fecal samples were collected and stored at -80°C for further analysis. This study was performed in accordance with the recommendations of the Guide for the Care and Use of Laboratory Animals (NIH, 8th Edition, 2011). This protocol was approved by the Southern Medical University review board.

### Models of Myocardial Infarction

The mice subjected to surgery in this study were first anesthetized using an intraperitoneal injection of a mixture of xylazine (5 mg/kg) and ketamine (100 mg/kg). After anesthesia, the trachea was intubated for mechanical ventilation (inspiration/expiration ratio: 1:3, 120 strokes/min), the left thorax was opened, and left coronary artery ligation was performed to induce MI. Successful ligation was verified in an ST-segment elevation using an electrocardiogram. The sham-operated mice were subjected to the same treatment without ligation. At 1 week after the operation, cardiac function was measured using echocardiography, and fecal samples were collected for further analysis. In addition, the mice were sacrificed using an overdose of pentobarbital followed by cervical dislocation. Heart weight, lung weight and body weight were then measured.

### Echocardiography

Echocardiography was performed in anesthetized (2% isoflurane) mice using a VEVO2100 system (Visual Sonic, North American) as previously described. The LV end-diastolic diameter (LVEDD) and LV end-systolic diameter (LVESD) were measured in M-mode, in which EF (ejection fraction), FS (fractional shortening), and LVESD are used to determine contractility and the size of the cardiac chambers. CO (cardiac output) indicates the volume of blood pumped from the left ventricle per minute, and SV (stroke volume) indicates the volume of blood pumped from the left ventricle per beat.

### DNA Extraction, 16S rRNA Gene Amplification, Bioinformatics and Statistical Analysis

Bacterial genomic DNA was extracted from fecal samples using a Fecal Total DNA EXTRACT kit (BioEAsy, Shenzhen). The 16S rRNA genes were amplified according to a previously described protocol (Liu et al., 2017). The sequences were deposited in the European Nucleotide Archive (ENA) under the accession number ERS1812205. The V4 hypervariable region was sequenced using the PE100 sequencing strategy. We used the BIPES pipeline to process the raw sequences (Zhou et al., 2011). First, the barcode primers were trimmed and filtered if they contained ambiguous bases or mismatches in the primer regions according to the BIPES protocol. Second, we removed any sequences with more than one mismatch within the 40–70 bp region at each end. Third, we used 30 Ns to concentrate the two single-ended sequences for the downstream sequence analyses. A detailed description of these methods was previously reported (Liu et al., 2017). Third, we performed UCHIME (implemented in USEARCH, version 6.1) to screen out and remove chimeras in the *de novo* mode (using –minchunk 20 –xn 7 –noskipgaps 2) (Edgar and Flyvbjerg, 2015).

All subsequent analyses were performed using QIIME (1.9.1). The sequences were then clustered to an operational taxonomic unit (OTU) using USEARCH with default parameters (USERACH61). The threshold distance was set to 0.03. Hence, when the similarity between two 16S rRNA sequences was 97%, the sequences were classified as the same OTU. QIIME-based alignments of representative sequences were performed using PyNAST, and the Greengenes 13_8 database was used as the template file. The Ribosome Database project (RDP) algorithm was applied to classify the representative sequences into specific taxa using the default database (Edgar and Flyvbjerg, 2015). The Shannon and PD_whole_tree indices were used to analyze alpha diversity, and a principal coordinate analysis (PCoA) analysis was performed using QIIME based on the UniFrac distance (Caporaso et al., 2010). Beta-diversity analyses were performed based on the UniFrac distance, and Adonis was used to estimate the amount of dissimilarity in microbial compositions between groups and to describe the association between variation in the gut microbiome and heart functions. We used LEfSE (linear discriminant analysis effect size) to identify features that differed between the groups (Segata et al., 2011). The threshold on the logarithmic LDA score for discriminative features was set to 2.0. All statistical analyses were performed using SPSS 20.0.

## Results

### Cardiac Function in Exercise-Trained Mice after Left Coronary Artery Ligation

**FIGURE 2:**
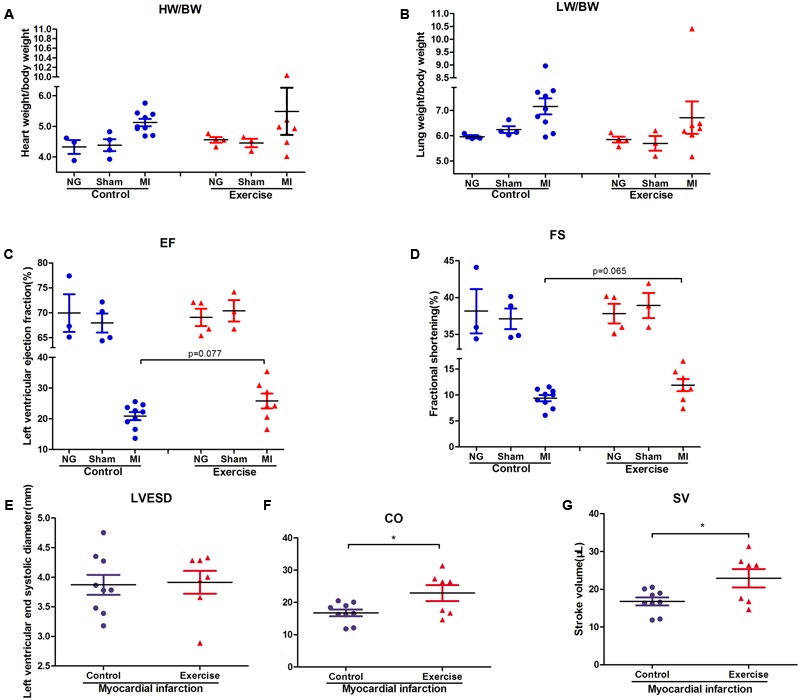
Cardiac function in exercise-trained mice after myocardial infarction. The ratio of heart weight/body weight and lung weight/body weight in mice subjected to left coronary artery ligation **(A,B)**, and the results of echocardiography in post-MI mice, including **(C)** ejection fraction (EF), **(D)** fractional shortening (FS), **(E)** left ventricular end-systolic diameter (LVESD), **(F)** cardiac output (CO) and **(G)** stroke volume (SV). ^∗^*P* < 0.05 compared to the corresponding groups.

To explore the effect of physical exercise training on MI mice, C57BL/6J mice were randomly divided into six groups. The ratios of heart weight/body weight (HW/BW) and lung weight/body weight (LW/BW) were measured and calculated. However, there were no significant differences in these ratios between exercise-MI mice and control-MI mice (**Figures 2A,B**). Exercise-MI mice showed an upward trend in EF and FS (**Figures 2C,D**) but no significant change in LVESD (**Figure 2E**). As expected, cardiac output (CO) and stroke volume (SV) were higher in exercise-MI mice than in control-MI mice (*P* < 0.05; **Figures 2F,G**). Taken together, these results demonstrate that moderate-intensity exercise training benefited cardiac functions in MI mice.

### Physical Exercise and Myocardial Infarction Affect the Gut Microbiota

**FIGURE 3:**
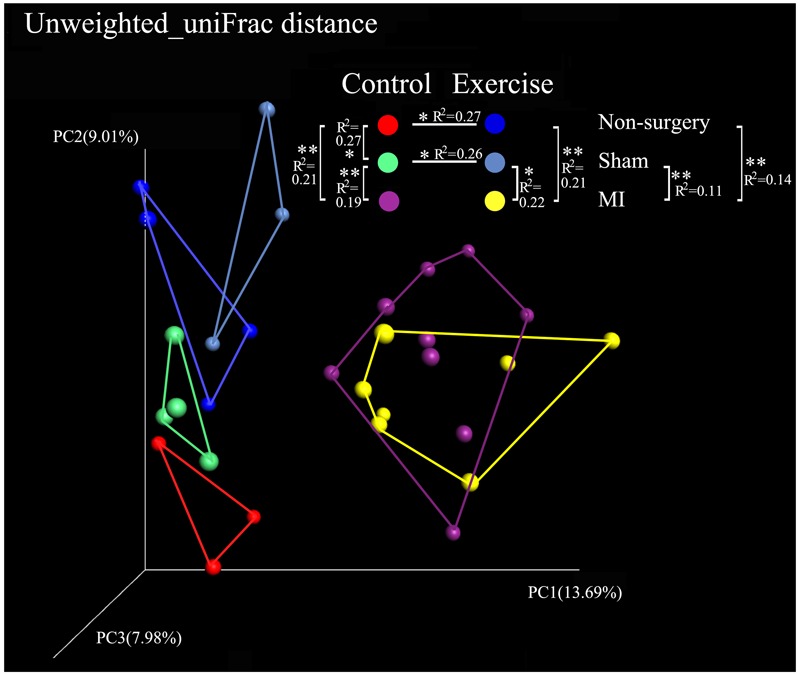
Comparison of the microbial community in the non-surgery, sham and MI mice based on uniFrac distances obtained in a PCoA analysis. Unweighted_uniFrac distances among the exercise-trained group (blue), the negative control group (red), the sham-control group (green), the sham-exercise-trained group (light blue), the MI-exercise-trained group (yellow) and the MI-control group (purple).

Alpha diversity was calculated using QIIME by the PD_whole_tree and the Shannon indices. The PD_whole_tree index represents the phylogenetic diversity of the community structure, while the Shannon index represents both the richness and evenness of its species diversity. Our results demonstrated that the values of the alpha diversity indices were lower in the negative control mice than in the exercise-trained mice, suggesting that there was more bacterial diversity in the exercise-trained group than in the negative control group. To identify differences in the composition of the bacterial community structure between the two groups, a PCoA was performed in QIIME. Fecal samples clustered into two distinct sets in the non-surgery and sham groups, suggesting that they had different bacterial community structures. This was further confirmed by the finding that the unweighted uniFrac values in the Adonis results were significantly different between these two groups (*R*^2^ = 0.27, *P* < 0.05 in non-surgery group; *R*^2^ = 0.26, *P* < 0.05 in sham group) (**Figure 3**). While exercise may also affect the community composition of the gut microbiota in the MI group, a beta-diversity analysis of the uniFrac distance was performed in a PCoA analysis and revealed clustering between the exercise and non-exercise samples obtained from the MI groups. In addition, Adonis analysis revealed no significant differences between these two groups. We also found that MI significantly affected the gut microbiota by comparison of the MI group with the sham (*R*^2^ = 0.22, *P* < 0.05) or non-surgery group (*R*^2^ = 0.21, *P* < 0.01) among the exercise-trained mice, and similar findings were observed for the sedentary mice (MI group vs. sham group: *R*^2^ = 0.19, *P* < 0.01; MI group vs. non-surgery group: *R*^2^ = 0.21, *P* < 0.01) (**Figure 3**). These results indicated that the gut microbiota was more strongly shifted by MI than by physical exercise, and this may explain why there was no significant difference between the exercise and control groups that were both treated with MI.

**FIGURE 4:**
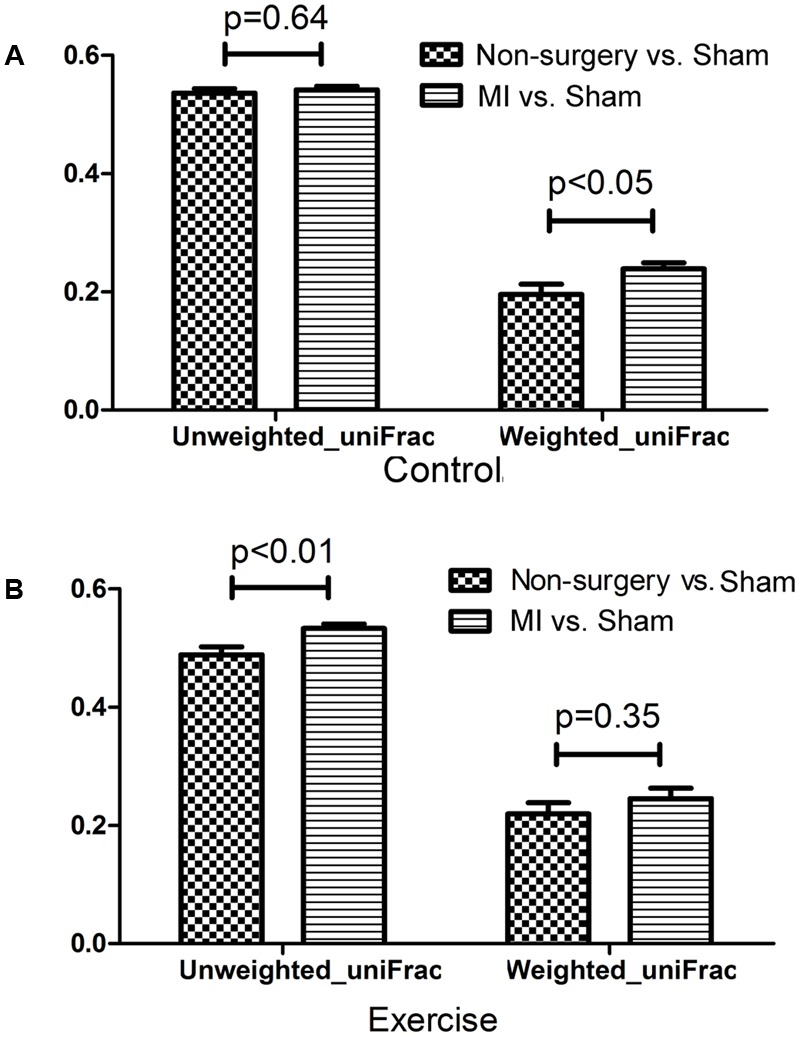
Dissimilarity in microbial community structure following MI between the non-surgery groups and the sham groups. UniFrac distances were determined to describe the dissimilarity between the non-surgery and sham groups and between the MI and sham groups in **(A)** non-exercise-trained and **(B)** exercise-trained mice (independent samples *t*-test).

Both left coronary ligation and sham surgery altered the gut microbiota in mice. To acquire a more accurate view of the changes that occurred in the gut microbial community of MI mice, we performed beta diversity analysis using weighted_uniFrac and unweighted_uniFrac distances to compare the differences between (non-surgery vs. sham) and (MI vs. sham) groups for both the exercise-trained and non-exercise-trained mice. The dissimilarity distance in the MI vs. sham comparison was significantly higher than that in the non-surgery vs. sham comparison, further suggesting that the MI groups experienced greater changes in the gut microbiota than were observed in the sham-operated mice and those not treated with surgery (**Figure 4**).

**FIGURE 5:**
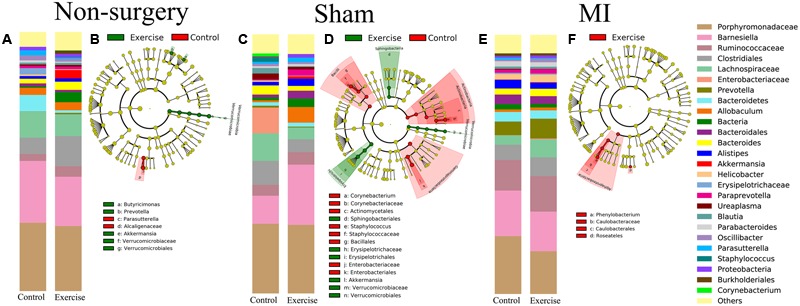
Relative abundance and LEfSE in the bacterial community structure in the non-surgery, sham and MI groups. Relative abundance of major genera in bacterial communities among the **(A)** non-surgery, **(C)** sham and **(E)** MI groups. Differential features were selected according to LEfSE between the exercise-trained and control mice in the **(B)** non-surgery, **(D)** sham and **(F)** MI groups.

We also analyzed the differences in composition between the exercise-trained and control mice in the non-surgery, sham and MI groups (**Figures 5A,C,E**). Then, we performed a LEfSE analysis to determine which bacterial taxa were distinct within the two groups. The results of a LDA effect size analysis showed that *Butyricimonas, Prevotella*, and *Akkermansia* were significantly more abundant in exercise-trained mice, while *Parasutterella* was mostly associated with the control group in the non-surgery groups (**Figure 5B**). *Erysipelotrichaceae, Sphingobacteriales*, and *Akkermansia* were significantly more abundant in the exercise-trained mice, while *Corynebacterium, Staphylococcus*, and *Enterobacteriaceae* were primarily associated with the control group for the sham groups (**Figure 5D**). Although a beta diversity test showed that there was a slight dissimilarity between the exercise-trained and non-exercise-trained MI-treated groups, a LEfSE analysis was still performed. The LEfSE analysis showed that there were more *Phenylobacterium* and *Roseateles* in the exercise-trained MI mice (**Figure 5F**).

### Association between Gut Microbial Community and Cardiac Function

**FIGURE 6:**
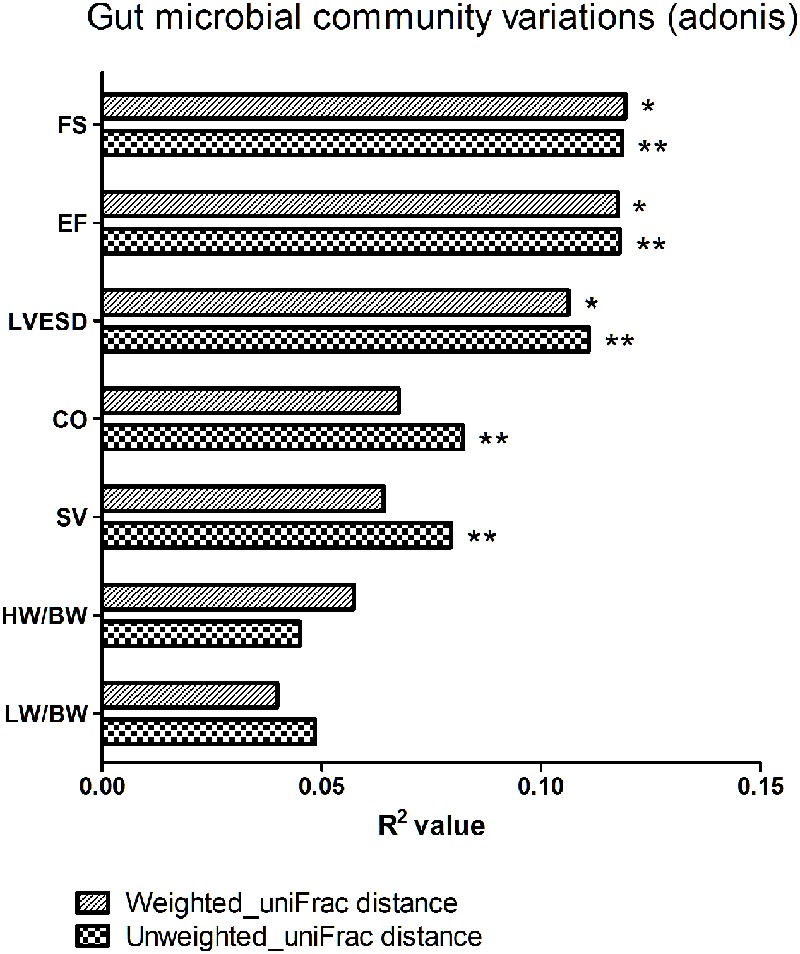
Beta diversity of the gut microbial community among various parameters of cardiac function. FS, fractional shortening; EF, ejection fraction; LVESD, left ventricular end-systolic diameter; CO, cardiac output; SV, stroke volume; HW/BW, the ratio of heart weight/body weight; LW/BW, the ratio of lung weight/body weight. The *R*^2^ value represents the results of Adonis. ^∗^*P* < 0.05, ^∗∗^*P* < 0.01.

We next explored the relationship between beta diversity in the gut microbial community and parameters associated with cardiac function. The results demonstrated that FS, EF, and LVESD (weighted uniFrac distance, *P* < 0.05; unweighted uniFrac distance, *P* < 0.01), in addition to CO and SV (unweighted uniFrac distance, *P* < 0.01), were significantly correlated with the gut microbiota in Adonis analysis (**Figure 6**).

**FIGURE 7:**
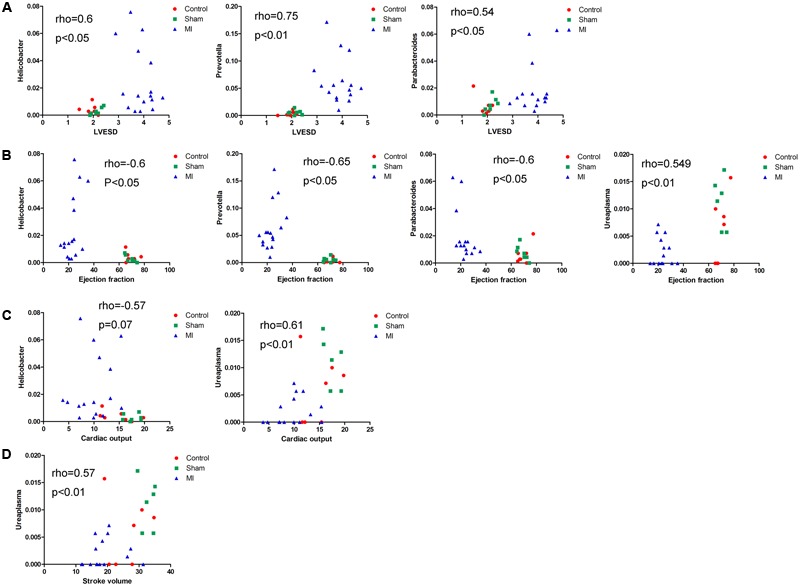
Correlations between cardiac function and the relative abundance of certain bacterial taxa. Correlations between the relative abundance of taxa and echocardiographic parameters: **(A)** left ventricular end systolic diameter, **(B)** left ventricular ejection fraction, **(C)** cardiac output, and **(D)** stroke volume (Spearman’s rank correlation).

**FIGURE 8:**
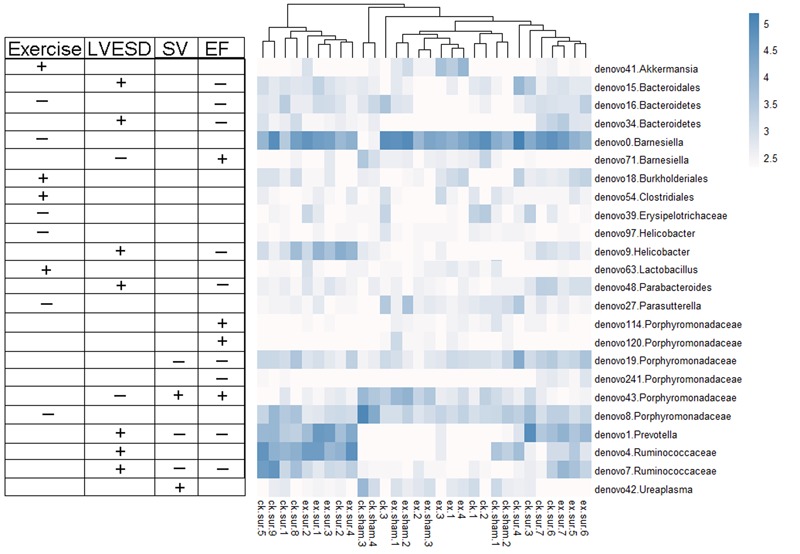
Heat map representing the important OTUs and their relationships with heart function and exercise. A total of 24 key variables were found to be significantly correlated with exercise or cardiac function (Spearman’s rank correlation). The symbols “+” and “-” represent positive and negative correlations among exercise, LVESD, SV, and EF.

We next explored the functions of and relationships between key variables and cardiac functions. The data are shown as a scatter plot (**Figure 7**), which indicates that while there was a significantly positive correlation between *Helicobacter, Prevotella*, and *Parabacteroides* and LVESD, all three of these genera were negatively correlated with EF. *Helicobacter* was also negatively correlated with CO, while *Ureaplasma* was positively correlated with EF, CO, and SV. Fifteen OTUs were significantly correlated with a group of features of heart function, including LVESD, EF, and SV (**Figure 8**). Three OTUs in Bacteroidetes appeared to be crucial based on their relationships with EF and LVESD. Two OTUs in *Barnesiella* suggest that this be a controversial genus because denovo0 was decreased in the exercise-trained mice and denovo71 was increased in mice with better cardiac function. Among *Prevotella, Helicobacter*, and *Parabacteroides*, only one OTU in each genus was positively correlated with worse cardiac function. Additionally, the classifications of all 3 OTUs was unclear because we were unable to determine their species. Two OTUs in Ruminococcaceae were negatively correlated with heart function. We also found that while 5 OTUs in Porphyromonadaceae were closely associated with cardiac function, the function of this family was biased. Moreover, 4 OTUs, including denovo1 in *Akkermansia*, denovo18 in Burkholderiales, denovo54 in Clostridiales and denovo63 in *Lactobacillus*, were increased in the exercise-trained groups. Among these taxa, *Akkermansia*, Clostridiales and *Lactobacillus* are traditionally regarded as prebiotics. Taken together, these findings suggest that intestinal bacteria and cardiac function are associated with each other to some extent. However, experiments performed using germ-free animals would further confirm the roles of these bacteria.

## Discussion

In this manuscript, we provide evidence that physical exercise affects cardiac function in MI mice and that it can modulate the gut microbiome. Recently, the connection between CVD and the gut microbiome has emerged and is a rapidly developing topic. Other studies have also shown that exercise alters the intestinal microbiota (Petriz et al., 2014; Allen et al., 2015; Denou et al., 2016). It is plausible that exercise training could effectively prevent CVD by influencing the composition of the gut microbiota.

In the present study, we found that CO and SV were higher in exercise-trained than in control MI mice. Moreover, compared to control MI mice, exercise MI mice tended to have higher EF and FS, although these differences were not significant. Of particular note is the finding that exercise was previously shown to exert cardioprotective effects in MI mice by inhibiting oxidative stress, ameliorating inflammatory injury, increasing the expression of VEGF (vascular endothelial growth factor) and reducing MI size (Ding et al., 2005; Wu et al., 2009; Salimeh et al., 2013). Thus, the alteration of the gut microbial composition by physical exercise might be a novel explanation for the beneficial effects exerted by exercise in MI.

To further explore our hypothesis, we performed a PCoA in QIIME. We found that gut microbiota compositions were closely related with exercise in both the non-surgery and the sham groups, but there was no significance in the MI groups. Additionally, our results suggest that the MI mice harbored a distinct gut bacterial community structure, as demonstrated by the fact that the dissimilarities observed between the negative control group and the sham group were significantly smaller than those between the MI and sham groups in the weighted uniFrac and unweighted uniFrac distance analyses.

One potential reason that there was no significant difference between the MI groups is that MI severely influences cardiac function and leads to changes in intestinal blood perfusion and the intestinal microenvironment, and these alterations might disguise the influence of exercise. The underlying mechanism that connects CVDs and the gut microbiota are being extensively researched because most heart diseases are accompanied by reduced left ventricular EF, and reduced EF can eventually lead to intestinal congestion. This pathophysiology would change the microenvironment of the intestines by inducing effects such as hypoxia, ischemia, hypercapnia and a redox state (Alverdy et al., 2005). All of these alterations can influence the microbial composition and structure, resulting in the generation and release of more toxic products into the circulation. These release factors could then impair heart and renal function. Renal injuries reduce the ability to eliminate toxins and aggravate edema, which is harmful to the cardiac system and vice versa. As a consequence, modulating the gut microbiome might be a potential therapeutic strategy for improving cardiac function. Intriguingly, in one experimental study, the administration of vancomycin in mice alleviated heart failure by changing the gut microbiome (Lam et al., 2012). Additionally, probiotics have also been shown to exert beneficial effects in mice with heart failure (Gan et al., 2014). These observations provide insight into how CVDs regulate the microbiome and provide descriptions of the profiles of the intestinal microbiota under different morbid statuses.

In our study, we found that *Butyricimonas* and *Akkermansia* were significantly enriched in the exercise-trained mice, suggesting that they might exert beneficial effects before CVD development. In addition, there was also significantly more *Akkermansia* in sham exercise-trained mice than in sham-operation mice. It was previously shown that *Butyricimonas* species are significantly associated with plasma glucose levels in patients with insulin resistance (Moreno-Indias et al., 2016). Moreover, Zhao et al. (2017) reported that *Akkermansia* species exert anti-inflammatory effects in mice and could be a potential target for combating metabolic syndrome and obesity in future studies (Anhe et al., 2016; Leal-Díaz et al., 2016). Therefore, the microbiota could be modified by exercise through the mechanisms described above, resulting in the amelioration of insulin resistance and exerting anti-inflammatory effects. In fact, these effects are commonly thought to play protective roles in CVDs. In exercise-trained MI mice, the relative abundance of *Phenylobacterium* and *Roseateles* was higher than that observed in the corresponding control MI mice. These bacteria were reported to reside primarily in the surrounding environment. However, few reports have explored the effects and roles of these bacteria in heart disease (Singleton et al., 2016). A possible explanation for the alterations observed in this study is that impaired cardiac function results in intestinal ischemia and changes in the composition of the microbiota and vice versa. In addition, the MI model was induced by coronary ligation, in contrast with spontaneous MI, which is typically accompanied by risk factors such as diabetes, hyperlipidemia, or hypertension. Thus, different results could be observed in spontaneous MI. Nevertheless, whether the alterations observed in this study play roles in the cardiovascular system requires further investigation.

We also explored the correlation between the gut microbiota and echocardiography. The results indicated that *Helicobacter* was positively correlated with LVESD and significantly and negatively correlated with EF and CO. Currently, our understanding of *Helicobacter* is not limited to *Helicobacter pylori* in gastric ulcers in that emerging evidence indicates that *H. pylori* might also participate in coronary artery disease (Hughes, 2014; Sun et al., 2016). Thus, these bacteria might be a potential area of further study. We also found that *Prevotella* and *Parabacteroides* were negatively correlated with EF and positively correlated with LVESD. *Prevotella* and *Parabacteroides* are both within Bacteroidetes, which contains species that are generally regarded as “good bacteria” because they are negatively correlated with metabolic syndromes and diabetes (Haro et al., 2016; Matziouridou et al., 2016). Nevertheless, among our OTUs, only one OTU in each genus was found to be positively correlated with worse heart function, and the classifications of these OTUs were unclear because we could not determine their species. Thus, these results do not contradict previous findings.

We subsequently focused on the OTUs that were closely related to exercise and cardiac function in mice. The results and the heat map clearly showed that EF, which is the most crucial indicator of cardiac function, was positively correlated with *Barnesiella.* This species is commonly regarded as a bacteria that participates in metabolic disease (Marietta et al., 2013; Al et al., 2017) and has been shown to be a crucial factor in immunological therapy (Daillère et al., 2016). However, the metabolic state of MI mice is different from that of patients, as the most common type of MI was caused by plaque rupture and was associated with various cardiovascular risk factors. This model of MI is most likely type-II MI, which is caused by coronary spasm or embolization (Thygesen et al., 2012). Therefore, these results may not have a broad scope of application. However, *Barnesiella* might be a good bacterium to use for treating or preventing type-II MI. Moreover, we identified 6 OTUs in the Porphyromonadaceae family that were closely associated with MI. These included 3 OTUs that were positively associated with EF values, 2 OTUs that were negatively associated with EF and only one OTU that was decreased in the exercise-trained group. Previous studies have suggested that some genera in this family are associated with diabetes, worse cardiac phenotypes and obesity (Clarke et al., 2013; Branchereau et al., 2016). In addition, previous findings suggested that Clostridiales is potentially involved in fatty acid metabolism (Chamkha et al., 2001), and it was found to be positively correlated with exercise. It is not difficult to imagine a bacteria that is capable of degrading fat, and this attractive ability might be applicable to the field of obesity, heart failure and atherosclerosis.

However, there are some limitations and drawbacks to consider in this study. First, exercise increases food intake, and food consumption would also change the gut microbiota. In our experiment, the mice were provided free access to food and water, and even mild or moderate exercise is likely to increase food intake. However, after 4 weeks of exercise, the mice had a 1-week resting period before fecal samples were collected. We found that *Akkermansia* were increased after exercise in both the control and sham groups. Previous studies indicated that fasting enriched the abundance of *Akkermansia* (Sonoyama et al., 2009), whereas a high fat diet had the opposite effect (Singh et al., 2017). Hence, if food intake increases, *Akkermansia* would likely decrease. Therefore, the effect of exercise on shifts in the gut microbiota are stronger than those caused by exercise-driven increases in food intake. Second, the size of control group was small, and we therefore cannot completely exclude a cage effect. However, the mice in the study were reared in a homogenous environment and with a common genetic background, and this may to a certain extent compensate for this defect. In addition, a previous study has shown that the cage effect occurs over time within each cage and that this effect is inevitable. The previous PCoA results have revealed that mice with pre-existing gut microbial communities show microbiota similarities after 4 weeks, while our results demonstrated that the experimental effect resulted in greater microbiota differences (McCafferty et al., 2013). Thus, the experimental effect might be larger than the cage effect. Third, we did not demonstrate that certain bacteria have a direct effect on cardiac function, and we would need germ-free mice to do so. Fourth, the mechanism by which the bacteria modulate the immune system and endocrine secretion remains unknown. Further investigation is required to explore this issue.

## Conclusion

Our results show that the alterations in gut microbial structure that are caused by physical exercise are correlated with cardiac function in MI mice and that a strategy that involves modulating the microbiota using exercise might be an attractive method for treating CVDs.

## Author Contributions

Substantial contributions to the conception or design of the study or the acquisition, analysis, or interpretation of data used in the study: ZL, H-YL, HZ, QZe, QZh, WL, HR, and DX. Drafting the study or revising it critically for important intellectual content: ZL, H-YL, QZh, WL, HZ, QZe, HR, and DX. Final approval of the version to be published: DX, ZL, H-YL, QZh, WL, HZ, QZe and HR. Agreement to be accountable for all aspects of the study and ensuring that questions related to the accuracy or integrity of any part of the study are appropriately investigated and resolved: DX, HR, ZL, H-YL, QZh, WL, HZ, and QZe.

## Conflict of Interest Statement

The authors declare that the research was conducted in the absence of any commercial or financial relationships that could be construed as a potential conflict of interest.
